# Vitamin D for Clinical Diseases in Women: An Indispensable Factor in Medicine and Dentistry

**DOI:** 10.3390/jcm11113104

**Published:** 2022-05-30

**Authors:** Dario Calafiore, Leonzio Fortunato, Mario Migliario

**Affiliations:** 1 Physical Medicine and Rehabilitation Unit, Department of Neurosciences, ASST Carlo Poma, 46100 Mantova, Italy; 2 Dentistry Unit, Department of Health Sciences, University of Catanzaro “Magna Graecia”, 88100 Catanzaro, Italy; leo@unicz.it; 3 Dentistry Unit, Department of Translational Medicine, University of Eastern Piedmont, 28100 Novara, Italy

Vitamin D deficiency is a global health problem occurring in all age groups and in countries with both high and low levels of sunlight, and improving its role in nutrition could be considered as a public health priority [1,2].

Vitamin D is a generic name referring to both vitamin D2 (ergocalciferol, of vegetable origin) and vitamin D3 (cholecalciferol, synthesized in animal organisms); hence, these steroid hormones could be obtained from both diet (e.g., mushroom, salmon, mackerel, and herring) and dietary supplements. Nevertheless, the primary source of vitamin D is exposure of the skin to sunlight (skin synthesis of vitamin D) [1].

The vitamin D receptor (VDR) is a transcription factor regulating the expression of genes which mediate its biologic activity; thus, vitamin D has an autocrine and paracrine function, regulating cellular proliferation and differentiation, cell maturation, and the innate immune system [3]. In addition to regulating gene expression, vitamin D acts as a hormone and is involved in several metabolic pathways; its endocrine activity could stimulate calcium transport across the plasma membrane, promoting serum calcium and phosphate homeostasis [3].

The wide distribution of VDR allows a pleiotropic beneficial effect of vitamin D on different target tissues, such as bone, muscle, intestine, kidneys, etc. [4]. In this context, particularly in women, vitamin D deficiency showed to be related to an increased risk of fractures and falls [5,6,7]; osteoarthritis [8]; sarcopenia [9,10]; cardiovascular diseases [11]; malignancy [12,13]; neurological diseases [14,15,16]; COVID-19 [17]; poor oral health [18,19]; periodontal diseases, especially during pregnancy [20]; and temporomandibular disorders (TMD) [21].

However, to date, the most investigated action of vitamin D is on bone tissue It might support skeletal health, improving bone mineralization through increased intestinal calcium absorption, prevention of secondary hyperparathyroidism, and direct effects on osteoblast formation; therefore, it might exert a positive effect on bone strength, quality, and consequently, in reducing risk of fracture, counteracting osteoporosis [22,23,24].

Furthermore, vitamin D is also considered a key factor in the turnover of articular cartilage. Amirkhizi et al. hypothesized that vitamin D could have anti-inflammatory effects and immunomodulatory properties, thus modulating the expression of inflammatory biomarkers in osteoarthritis [25]. Additionally, a reverse relationship between serum levels of inflammatory markers such as IL-1b, TNF-a, hs-CRP, and NF-rB p65, as well as the severity of clinical symptoms and vitamin D status was detected [8].

At the same time, it has been shown that vitamin D deficiency can be associated with a significant higher risk of an impairment of muscle function and, when combined with overweight, is significantly associated with loss of muscle mass [9,10]. In fact, vitamin D serum levels should be investigated considering their significant correlation with a reduction in muscle strength and physical performance [6].

In this context, vitamin D deficiency represents a critical risk factor in the onset of sarcopenia and related disease, such as sarcopenic dysphagia [25], playing a key role in nutritional status and proteins synthesis [26]. Loss of muscle mass seems to be strictly related to the decrease in vitamin D receptor (VDR) expression, eliciting two different effects: (1) a non-genomic effect, such as the modulation of calcium channel activation, muscle contraction and mitochondrial function; (2) a genomic effect, up-regulating the nuclear expression of gene-coding contractile proteins and myogenic transcription factors [3,27].

A higher inflammatory status underpinning the vitamin D deficiency might also explain its role in neurological diseases, such as multiple sclerosis [14], stroke [28], and Parkinson disease [15]. These findings can support a possible role of vitamin D deficiency on brain health, notably for the risk of dementia and cognitive impairment; indeed, Navale et al. showed that hypovitaminosis D was associated with neuroimaging outcomes and a higher risk of dementia and stroke [29].

Furthermore, vitamin D is involved in regulating normal innate and adaptive immunity [30], and it has been shown to suppress pro-inflammatory, and stimulate anti-inflammatory cytokine formation [31], considering the rehabilitation needs of COVID-19 patients [32].

In light of recent evidence, recovered COVID-19 patients showed insufficient 25(OH)D3 serum levels, thus making vitamin D supplementation necessary for treating COVID-19 patients [17]. It should also be considered that vitamin D status has a clinical significance in the field of dentistry. In this scenario, several papers explored the relationship between hypovitaminosis D and poor oral health, but only a few clinical trials investigated the effects of vitamin D supplementation on oral status, as reported by a systematic review published in April 2022 by Ab Malik et al. [19]. However, we are aware that there is a high heterogeneity among oral health outcomes, considering the different parameters that might be investigated. Moreover, the correlation between serum levels of 25(OH)D3 and periodontitis has been widely investigated, with contrasting results, albeit most studies confirmed this hypothesis [33,34,35], particularly in pregnant women [18] or in those affected by breast cancer [20]. However, to date, although the study quality of the scientific literature has proven to have some limitations, vitamin D supplementation seemed to improve oral health status [19].

Vitamin D supplementation might be considered as a novel therapeutic option for the complex management of TMD that commonly include occlusal splints, extracorporeal shock wave therapy, laser therapy, and physical therapy [21,36,37,38,39]. As mentioned above, vitamin D has shown a potential effect on osteoarthritis and general joint pain [40]; thus, patients suffering from TMD have recently been investigated for their vitamin D serum levels. Kui et al. [21] reported that TMD patients with deficient levels of vitamin D (<30 ng/mL) are most likely to benefit from supplementation. In keeping with this scenario, further studies should better investigate the role that vitamin D might play in terms of pain relief and the functional improvement of patients who are affected by TMD.

In relation to the involvement of vitamin D in regulating normal innate and adaptive immunity, its contribution in the pathogenesis of premalignant oral conditions was recently explored [41,42]. Among them, lichen planus is known to be a chronic inflammatory disease, characterized by symmetrical and bilateral lesions in the oral mucosa, which can be uncomfortable or accompanied by burning and pain. Its etiology is not yet well understood; however, the pathogenesis seems to be related to the immune system’s T lymphocytes. Thus, the VDR presence on T lymphocytes and macrophages, and on the mature TCD8 + lymphocytes may explain the potential role of vitamin D in the management of premalignant oral conditions [41,42,43].

Taken together, the present scientific literature investigated the pleiotropic role of vitamin D in women in the last few years, but further research is still needed to deeply investigate the mechanisms underpinning the correlation between vitamin D and several diseases, in the fields of both medicine and dentistry.
